# Quantitative Microbial Risk Assessment of *E. coli* in Riverine and Deltaic Waters of Northeastern Greece: Monte Carlo Simulation and Predictive Perspectives

**DOI:** 10.3390/toxics13100863

**Published:** 2025-10-11

**Authors:** Agathi Voltezou, Elpida Giorgi, Christos Stefanis, Konstantinos Kalentzis, Elisavet Stavropoulou, Agathangelos Stavropoulos, Evangelia Nena, Chrysoula (Chrysa) Voidarou, Christina Tsigalou, Theodoros C. Konstantinidis, Eugenia Bezirtzoglou

**Affiliations:** 1 Laboratory of Hygiene and Environmental Protection, Faculty of Medicine, Democritus University of Thrace, 68100 Alexandroupolis, Greece; lvoltez@admin.duth.gr (A.V.); elpidagiorgi94@gmail.com (E.G.); kkalentz@med.duth.gr (K.K.); elisabeth.stavropoulou@gmail.com (E.S.); ctsigalo@med.duth.gr (C.T.); tconstan@med.duth.gr (T.C.K.); empezirt@yahoo.gr (E.B.); 2 School of Social and Political Sciences, University of Glasgow, Glasgow G12 8RT, UK; angelostavrop@gmail.com; 3 Laboratory of Social Medicine, Medical School, Democritus University of Thrace, 68100 Alexandroupolis, Greece; enena@med.duth.gr; 4 Department of Agriculture, School of Agriculture, University of Ioannina, 47100 Arta, Greece; xvoidarou@uoi.gr

**Keywords:** QMRA, public health, *E. coli*, dose–response model, Monte Carlo, river water, deltas, health risk, pathogens

## Abstract

This study presents a comprehensive Quantitative Microbial Risk Assessment (QMRA) for *Escherichia coli* in northeastern Greece’s riverine and deltaic aquatic systems, evaluating potential human health risks from recreational water exposure. The analysis integrates seasonal microbiological monitoring data—*E. coli*, total coliforms, enterococci, *Salmonella* spp., *Clostridium perfringens* (spores and vegetative forms), and physicochemical parameters (e.g., pH, temperature, BOD_5_)—across multiple sites. A beta-Poisson dose–response model within a Monte Carlo simulation framework (10,000 iterations) was applied to five exposure scenarios, simulating varying ingestion volumes for different population groups. Median annual infection risks ranged from negligible to high, with several locations (e.g., Mandra River, Konsynthos South, and Delta Evros) surpassing the World Health Organization (WHO)’s benchmark of 10^−4^ infections per person per year. A Gradient Boosting Regressor (GBR) model was developed to enhance predictive capacity, demonstrating superior accuracy metrics. Permutation Importance analysis identified enterococci, total coliforms, BOD_5_, temperature, pH, and seasons as critical predictors of *E. coli* concentrations. Additionally, sensitivity analysis highlighted the dominant role of ingestion volume and *E. coli* levels across all scenarios and sites. These findings support the integration of ML-based tools and probabilistic modelling in water quality risk governance, enabling proactive public health strategies in vulnerable or high-use recreational zones.

## 1. Introduction

Water quality is fundamental for human well-being and societal development, as highlighted by the UN’s Sustainable Development Goals (SDGs) for 2030, which stress the importance of protecting water resources and preventing pollution for various human activities [1,2,3]. Waterborne pathogenic microorganisms continue to pose a significant threat to public health globally, causing widespread enteric and diarrheal diseases, with a notable impact on vulnerable populations. The Global Burden of Disease study estimates that diarrheal diseases, which are strongly associated with inadequate water and sanitation infrastructure, caused 1.3 million deaths globally in 2015, including 0.5 million children under five [4,5]. Τhe United States records an estimated 4.3–16.4 million cases of acute gastroenteritis linked to public water systems annually, leading to thousands of hospitalizations and substantial economic costs [6].

*Escherichia coli* and enterococci serve as key indicators of fecal contamination in aquatic ecosystems, although it is recognized that no single indicator can perfectly predict the presence of all pathogens. Recreational activities in water are a major source of waterborne infections, with fecal pollution being the primary cause. *E. coli* is considered a reliable indicator due to its high concentration in mammalian feces, though its relationship with pathogen presence can be influenced by factors like dilution and water flow [7,8]. Beyond their role as quality indicators, pathogenic and antimicrobial-resistant strains of *E. coli* are also of growing concern within the One Health framework, as they can act as vectors for antimicrobial resistance genes (ARGs) [9,10,11].

In Greece, existing studies on aquatic ecosystems often focus on physicochemical parameters and lack systematic, long-term monitoring of microbial pathogen loads [12,13,14,15,16,17,18,19,20,21,22,23,24]. While some recent studies have reported the presence of *E. coli* in various surface waters, most of the findings are fragmented. A large-scale study on coastal waters in Eastern Macedonia and Thrace (EMT) confirmed that *E. coli* levels largely comply with European standards for “Excellent” quality [25,26,27,28].

QMRA is a four-phase process used to evaluate microbial risks in various media, including food and water [29,30,31,32]. The first step, hazard identification, involves identifying pathogens that may pose a health risk, along with physical conditions that influence their presence [33]. This is followed by exposure assessment, which quantifies the pathogenic agent in the exposure medium, considering factors like contamination sources and environmental processes.

The dose–response modelling phase integrates data from previous stages using mathematical tools and predictive models to estimate the probability of infection [33]. Finally, risk characterization combines all information to provide a comprehensive qualitative and quantitative risk assessment. This approach is widely used, with regulatory bodies like the European Food Safety Authority (EFSA) employing it for predictive risk analysis [34].

*E. coli* is a key indicator of purity in various aquatic ecosystems, including recreational waters, where its presence is monitored under directives like 2006/7/EC. This directive sets acceptable thresholds for *E. coli* concentrations, with an “Excellent” rating requiring levels below 250 CFU/100 mL, and “Good” or “Sufficient” ratings allowing up to 500 CFU/100 mL [35].

The main objective of this study was to perform a comprehensive Quantitative Microbial Risk Assessment (QMRA) for *Escherichia coli* in the riverine and deltaic ecosystems of Northeastern Greece. Specifically, we aimed to (i) quantify the probability of infection under different ingestion scenarios using Monte Carlo simulations; (ii) evaluate spatiotemporal variations in microbial water quality and their association with physicochemical parameters; and (iii) develop predictive models using Machine Learning (Gradient Boosting Regressor and Random Forest Regressor) to identify key environmental drivers of microbial risk. By integrating traditional QMRA with these emerging computational methodologies, this work presents an innovative framework for environmental health risk assessment. This framework contributes to enhanced water safety management by comparing the findings with existing European Directive (2006/7/EC) thresholds and providing data-driven policy recommendations for integrating risk assessment into water quality management and public health protection in the region.

## 2. Materials and Methods

### 2.1. Study Area and Sampling Campaign

The study was conducted in the geomorphologically diverse region of EMT, which is characterized by a mix of mountainous and coastal areas. This region includes the southern mountainous mass of the Rhodope mountain range, coastal lowlands, and major rivers. The EMT contains a variety of sensitive ecosystems, including cross-border rivers and wetlands protected under international conventions like Ramsar. Extended ecological and geographic information, including Natura 2000 sites, Ramsar wetlands, and detailed site descriptions, is provided in Supplementary Material S1.

**Figure 1:**
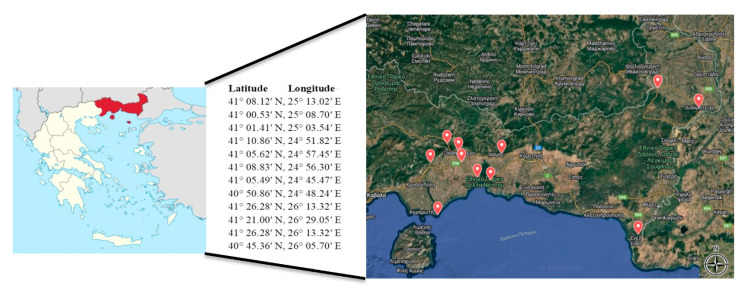
Sampling area and sampling points (two points on the map appear as one because they are very close).

Twelve inland and coastal sampling sites were selected across the Regional Units of Rhodopi and Xanthi, located near ecologically significant zones such as Lake Vistonida and the Kompsatos River (Figure 1). A total of 48 water samples were collected across four seasons (summer, autumn, winter, and spring) between 2022 and 2023. The selected locations were chosen as they are representative of diverse aquatic ecosystems, such as rivers and deltas, and allow for consistent seasonal monitoring.

At each site, field measurements and respective laboratory analyses were performed to assess a series of microbiological and physicochemical parameters, including *E. coli*, enterococci, *Clostridium perfringens*, pH, temperature, BOD_5_, and *Salmonella* spp. All sampling and analytical procedures followed standardized protocols to ensure data reliability.

### 2.2. Microbiological and Physicochemical Analyses

Microbial water quality indicators included *Escherichia coli*, enterococci, total coliforms, *Clostridium perfringens* (spores and vegetative forms), and *Salmonella* spp. Physicochemical parameters included pH, temperature, and biochemical oxygen demand (BOD_5_). Enumeration of *E. coli* was performed using membrane filtration and chromogenic agar following ISO 9308-1:2014. Enterococci were quantified according to ISO 7899-2:2000. Detection of *Salmonella* spp. was conducted via selective enrichment and biochemical confirmation based on ISO 19250:2010. *Clostridium perfringens* spores and vegetative forms were detected using culture-based methods standardized for anaerobic bacteria. Physicochemical analyses followed standard protocols for water quality assessment [36,37,38,39,40]. Results were expressed as colony-forming units (CFU/100 mL) or log-transformed values when appropriate. Quality control included duplicate analyses, blanks, and reference standards. (Full microbiological and physicochemical protocols, including media, reagents, incubation conditions, and confirmation steps, are detailed in Supplementary Material S2).

### 2.3. Data Analysis and Modelling

Descriptive statistics were calculated for all parameters, with normality tested via the Kolmogorov–Smirnov test. We used non-parametric methods where appropriate. Seasonal and spatial variations were evaluated with the Kruskal–Wallis test, and Spearman’s rank correlation coefficient was used to assess relationships between microbiological indicators and environmental variables [41,42,43].

We applied two supervised ML models, Random Forest Regressor (RFR) and Gradient Boosting Regressor (GBR), to predict *E. coli* concentrations based on environmental and microbiological inputs [44]. A 10-fold cross-validation strategy was implemented to ensure robust model performance [45]. The models were evaluated using standard metrics such as Root Mean Squared Error (RMSE), Mean Absolute Error (MAE), Mean Absolute Percentage Error (MAPE), and Coefficient of determination (R^2^) [46,47] (Supplementary Material S3. One-sample Kolmogorov–Smirnov test and modelling parameters as extracted from the Microsoft Azure Studio Classic). The performance of the best-performing models was interpreted using the Permutation Importance Method (PIM) [48,49,50]. PIM assesses the importance of each feature by measuring the drop in a model’s predictive performance when the values of a specific feature are randomly shuffled. This method provides a direct and reliable estimation of the influence of each variable on the model’s output. Unless stated otherwise, all statistical tests were performed at a significance level of 0.05.

Unless stated otherwise, statistical tests were performed at a significance level 0.05. All statistical analysis were performed by SPSS v.23 statistical software (SPSS, Chicago, IL, USA), Microsoft Excel and Microsoft Machine Learning Studio (https://learn.microsoft.com/en-us/azure/machine-learning/overview-what-is-azure-machine-learning?view=azureml-api-2; accessed on 1 March 2025).

### 2.4. QMRA Framework

QMRA comprises four stages (https://qmrawiki.org/node/8; accessed on 1 March 2025) to estimate the probability of infection associated with microbial hazards in a defined population. It includes four essential stages described above: hazard identification, exposure assessment, dose–response modelling, and risk characterization [51]. Each component was systematically applied in the current study for Escherichia coli in surface waters used for recreational activities in river deltas and sampling points of the current research [52,53].

#### 2.4.1. Hazard Identification

The first step of the QMRA framework involved identifying *E. coli* as the primary pathogen of concern, given its global prevalence in recreational waters and its dual role as a fecal contamination indicator and a potential pathogen. We assumed that a proportion of *E. coli* strains could be pathogenic, based on literature estimates suggesting that 0.005% to 0.1% of strains in surface water may be infectious to humans [54,55,56]. This selection was also supported by the results of our previous study on recreational waters in the coastal areas of EMT [25]. The use of *E. coli* is consistent with its role as a key quality indicator in EU Water Directives.

#### 2.4.2. Exposure Assessment

Exposure assessment quantifies the frequency, magnitude, and contact route with the microbial hazard. In this study, exposure scenarios were designed to reflect varying levels of incidental ingestion during recreational activities. Five ingestion volumes were modelled, representing different age groups and activities:

10 mL: Low-exposure scenario: incidental ingestion during wading, splashing, or minimal contact activities;

15 mL: Elderly-specific scenario: moderate incidental ingestion typical among elderly bathers engaging in limited immersion;

20 mL: Children-specific scenario: typical ingestion for young children playing near shore with frequent hand-to-mouth contact;

30 mL: Moderate-exposure scenario: partial head immersion during swimming or recreational activities;

50 mL: High-exposure scenario: full head immersion or active swimming where mouth and nose are submerged repeatedly.

The ingested dose was calculated using the following Equation (1):

(1) $$Dose\left(CFU\right)=C\times V\times IF$$

where

C = *E. coli* concentration (CFU/mL);

V = ingested volume (mL);

IF = infectious fraction of *E. coli* (assumed 0.01).

The assumed *E. coli* infectious fraction (IF = 0.01) corresponds to conservative estimates from relevant literature on the proportion of potentially pathogenic *E. coli* in surface waters [56,57,58,59]. This parameter was also probabilistically treated in the Monte Carlo simulation to encapsulate respective uncertainty.

#### 2.4.3. Dose–Response Modelling

The probability of infection following exposure was modelled using the Beta-Poisson dose–response model (Equation (2)), a well-established approach for microbial pathogens [60]:

(2) $$P_{inf}=\;1-{\left(1+\frac{d}{\beta }\right)}^{-a}$$

where

P_inf_ = probability of infection per exposure;

D = ingested dose (CFU);

α, β = model parameters (pathogen-specific).

Parameters were adopted from [60] for enteropathogenic *E. coli*:

α = 0.21, β = 2.11 × 10^6^ [61].

#### 2.4.4. Risk Characterization

**Figure 2:**
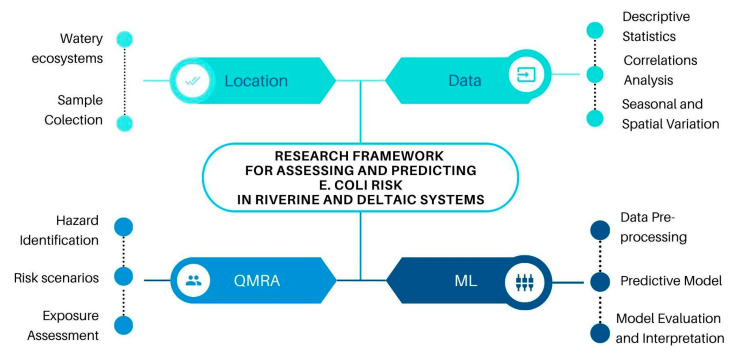
Research flow diagram.

In this stage, the cumulative annual probability of infection per year is estimated, encompassing the yearly number (n) of exposure events per year [62] Equation (3):

(3) $$P_{annual}=1-{\left(1-P_{inf}\right)}^{n}$$

where n varied depending on the assumed frequency of recreational water use (e.g., 10–50 exposures/year), we used a Monte Carlo simulation with 10,000 iterations to account for the natural variability and uncertainty of the input parameters, such as *E. coli* loads, ingestion volumes, and the infectious fraction [63,64,65]. This probabilistic analysis produced risk estimates with confidence intervals, enabling scenario-based comparisons. Furthermore, a sensitivity analysis was conducted using Partial Rank Correlation Coefficients (PRCC) to identify the most influential parameters affecting risk outcomes. These included the ingestion volume, *E. coli* concentration, and the infectious fraction [60,66,67,68]. This analysis, combined with an uncertainty analysis, helped us assess the robustness of our estimated contamination risks. Figure 2 depicts the research framework developed and applied in the current study.

## 3. Results

### 3.1. Microbiological and Physicochemical Analyses and ML Approach

The normality of the main microbiological and physicochemical parameters was assessed using the Kolmogorov–Smirnov test. Results showed that temperature (*p* = 0.221) and pH (*p* = 0.981) followed a normal distribution (*p* > 0.05). Conversely, total coliforms (*p* = 0.003), *E. coli* (*p* = 0.001), enterococci (*p* < 0.001), and BOD_5_ (*p* = 0.022) did not meet the normality criterion (*p* < 0.05), indicating a significant deviation from a normal distribution. Since most variables did not follow a normal distribution, non-parametric statistical methods were applied in subsequent analyses (Supplementary Material S3).

Based on a set of N = 48 samples, descriptive statistics for all six parameters are presented, with spatiotemporal variability of physicochemical parameters further illustrated in the Supplementary Material S3. The mean temperature was 15.5 °C, ranging from 7.2 °C to 25.9 °C, while the mean pH was 7.49, ranging from 6.54 to 8.62. For the microbiological parameters, the median values were 400.0 CFU/100 mL for total coliforms, 78.5 CFU/100 mL for *E. coli*, and 54.5 CFU/100 mL for enterococci. These parameters exhibited high positive skewness, with most observations clustered at lower values but with significant variations up to maximum concentrations of 1300.0 CFU/100 mL for total coliforms and 500.0 CFU/100 mL for both *E. coli* and enterococci, indicating periods of higher contamination. The full descriptive statistics can be found in Table 1.

Microbiological indicators exhibited strong spatial and seasonal variability. *E. coli* concentrations ranged from 0 to 500 CFU/100 mL (median 78.5), with peaks during summer and autumn at sites downstream of agricultural and urban discharges. Enterococci concentrations (median 54.5 CFU/100 mL) followed similar patterns, while total coliforms were consistently higher, indicating diffuse fecal pollution.

**Figure 3:**
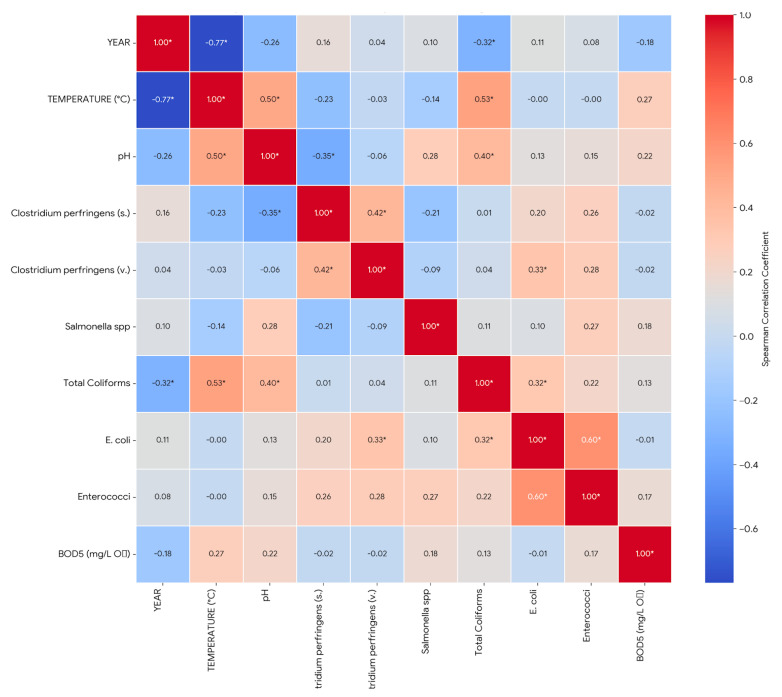
Heatmap of correlations between microbiological and physicochemical parameters. * Correlation is significant at the 0.05 level.

A correlation heatmap (Figure 3) revealed strong positive associations between the microbial indicators, with *E. coli* showing robust correlations with both enterococci (ρ = 0.60) and total coliforms (ρ = 0.57). The Spearman analysis provided additional insights, showing a moderate positive correlation between temperature and all key microbial indicators (total coliforms, *E. coli*, and enterococci), which is consistent with the understanding that warmer temperatures enhance microbial growth in aquatic systems (Full descriptive statistics and seasonal/spatial analyses are provided in Supplementary Material S4). Additionally, pH showed a positive correlation with temperature (r = 0.50) but a negative correlation with the spore-forming forms of *C. perfringens*, suggesting that higher pH values may be associated with lower concentrations of these microorganisms. The strong positive relationship between *E. coli* and enterococci (*p* = 0.60) reinforces their complementary use in assessing microbial burden and potential health risks.

**Figure 4:**
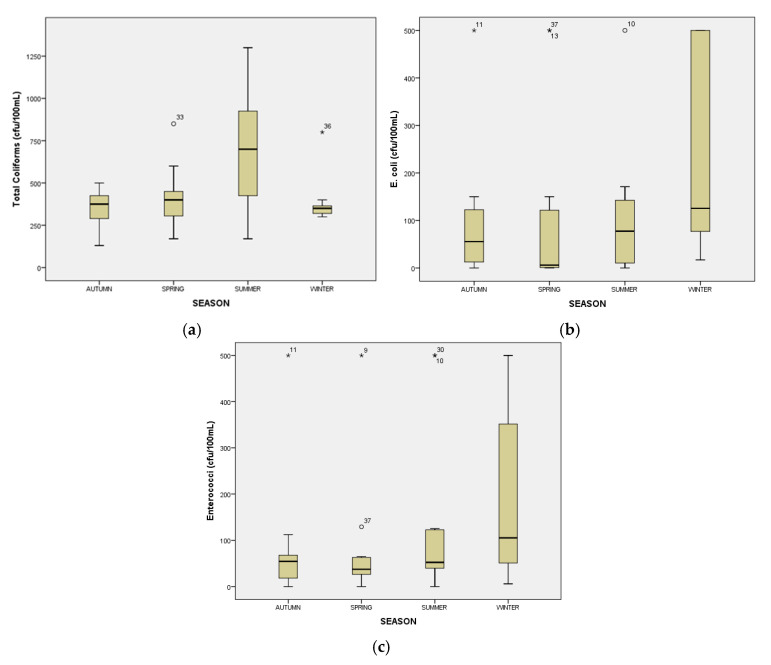
Seasonal variation in total coliforms (**a**), *E. coli* (**b**) and enterococci (**c**). (The box lines indicate the lower and upper quartiles; the central line is the median. The whiskers show the minimum and maximum values. Circles represent outliers, and asterisks represent extreme outliers).

**Figure 5:**
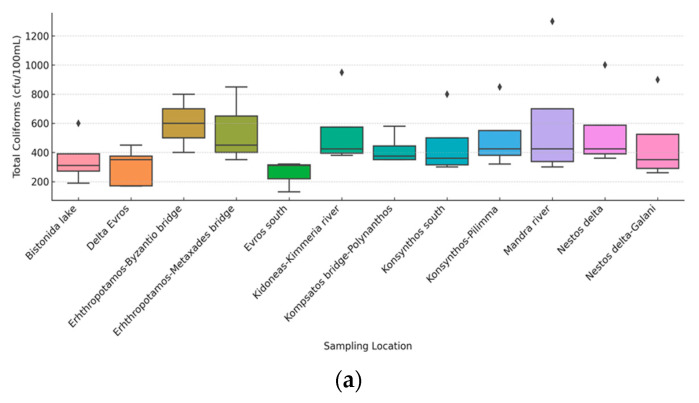

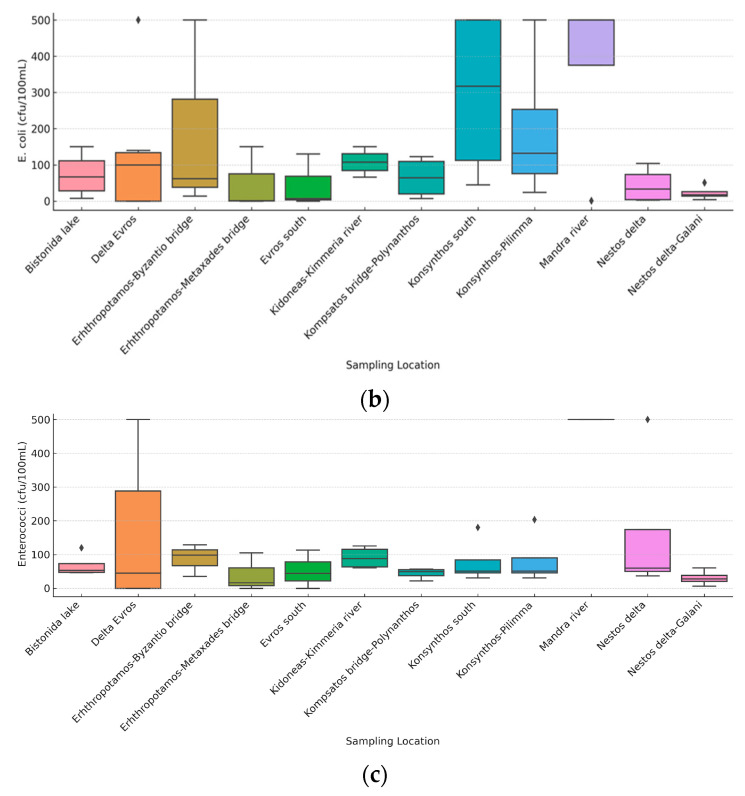
Spatial variation in total coliforms (**a**), *E. coli* (**b**) and enterococci (**c**). (The box lines indicate the lower and upper quartiles; the central line is the median. The whiskers show the minimum and maximum values.

Figure 4 and Figure 5 below illustrate the seasonal and spatial variation in total coliforms, *E. coli*, and enterococci concentrations between seasons and sampling points. Seasonal variations were observed only for total coliforms, as the applied Kruskal–Wallis test showed statistically significant differences among seasons [H(4) = 15.85, *p* = 0.001]. In contrast, no statistically significant seasonal differences were found for *E. coli* [H(4) = 5.363, *p* = 0.147] or enterococci [H(4) = 4.132, *p* = 0.248]. Furthermore, the Kruskal–Wallis test indicated no statistically significant differences in total coliforms, *E. coli*, or enterococci concentrations among all sampling locations [H(12) = 14.969, *p* = 0.184; H(12) = 12.169, *p* = 0.351; H(12) = 16.354, *p* = 0.128, respectively].

A significant seasonal variation was observed, with the highest concentrations in summer, indicating an increased microbial load during this period. The seasonal variation was statistically significant (Kruskal–Wallis test, *p* = 0.001).

While total coliforms showed a clear seasonal trend, the variations in *E. coli* and enterococci concentrations were not statistically significant (*p* = 0.147 and *p* = 0.248, respectively).

Concentrations for all three indicators showed a heterogeneous distribution across sampling areas, with noticeable fluctuations and extreme outliers, particularly for *E. coli* and total coliforms. In contrast, enterococci had a more compact distribution with less variability. For example, while the Kosynthos River showed significant variation in *E. coli*, Vistonida Lake had isolated high values of total coliforms, possibly indicating specific local contamination sources. However, these spatial differences were not statistically significant, reinforcing the need for further investigation with an enhanced sampling design (Figure 5).

Table 2 corresponds to modelling metrics results after 10-fold Cross-Validation. Both models demonstrate excellent performance, with very high R^2^ values (close to 1), indicating that they explain a substantial proportion of the variation in *E. coli* concentration.

The RFR model exhibits a slightly better R^2^ (0.9321 vs. 0.9113), which explains a larger proportion of the variation in *E. coli* concentration. However, the GBR has significantly lower RMSE and MAE, indicating smaller mean prediction errors. The MAPE and SMAPE values are also lower for GBR. This suggests that, although RF may have a slightly better overall “explanation” of the variation (R^2^), GBR is more accurate in its absolute and percentage error predictions.

**Figure 6:**
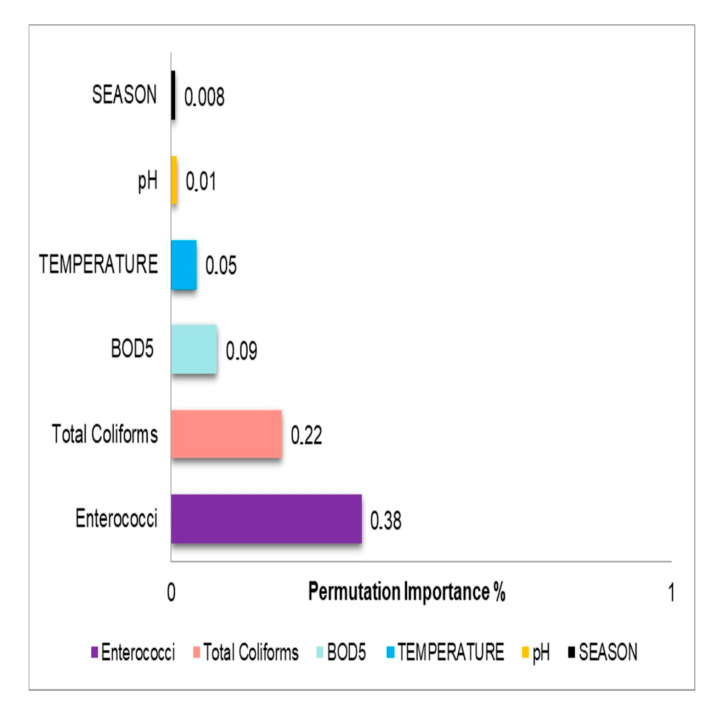
Permutation Importance for GBR.

Permutation Importance is a model-agnostic technique that measures a feature’s importance by observing how much the model’s performance decreases when the feature’s values are randomly shuffled. A higher importance score indicates the feature is more critical for the model’s predictive accuracy. Figure 6 visualizes the relative importance of each variable for the GBR model in predicting *E. coli* concentration.

For the GBR model used to predict *E. coli* concentrations, the analysis revealed that enterococci, total coliforms, and BOD_5_ were the most influential variables, indicating their substantial impact on the model’s predictive accuracy. Conversely, Temperature and pH exhibited lower relative importance, although they still contributed positively to the model. Variables such as Season, *Salmonella* spp., *Clostridium perfringens* (vegetative and spores), and Year had negligible or near-zero contributions, and thus did not significantly influence the model’s predictive ability.

### 3.2. QMRA Framework

**Figure 7:**
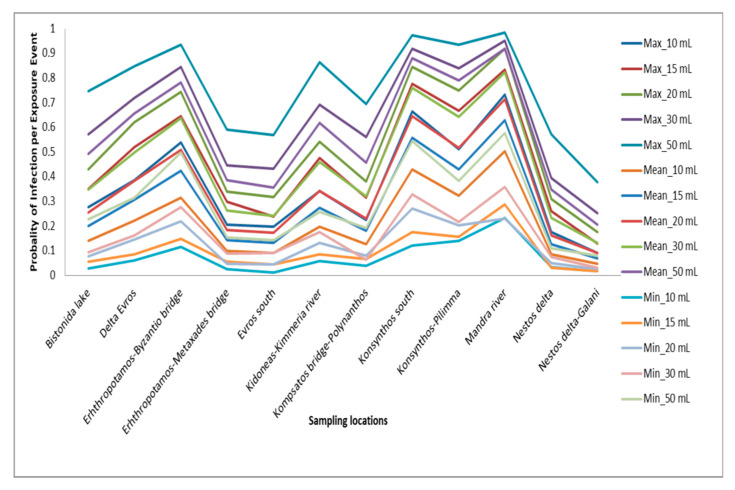
Minimum, mean and maximum infection risk by scenario and location.

Figure 7 depicts the probability of infection per exposure event for *E. coli* across all sampling locations and five ingestion scenarios (10–50 mL). It considers minimum, mean, and maximum risk estimates, which were derived using Monte Carlo simulations based on site-specific *E. coli* concentrations and the dose–response model recommended for enteric pathogens.

The probability of infection per exposure event to the pathogenic microorganism *Escherichia coli* was estimated for five realistic scenarios of recreational water ingestion:

10 mL: Low-exposure scenario (e.g., splashing or incidental contact);

15 mL: Elderly-specific scenario (moderate intake with increased susceptibility);

20 mL: Children-specific scenario (higher susceptibility and behaviour-specific intake);

30 mL: Moderate-exposure scenario (e.g., swimming);

50 mL: High-exposure scenario (e.g., swimmers with full head immersion).

Combined with the observed *E. coli* concentrations at 12 sampling locations, the calculated probability of infection systematically increases with the volume of ingestion (dose dependence), confirming the importance of the total ingested microbial dose in infection occurrence. In low-exposure scenarios (10–15 mL), the average probability of infection ranges between 0.05 and 0.30, with lower values in locations such as Nestos Delta–Galani and Vistonida Lake. In contrast, in moderate to high-exposure scenarios (30–50 mL), the probabilities of infection in areas such as Konsynthos South, Mandra River, and Erhthropotamos–Byzantio Bridge exceed 0.8–0.95, indicating a near certainty of infection per exposure.

Taking into account the WHO reference limit of 1 case of infection per 10,000 people per year (10^−4^), it is evident that even in the most favourable scenario (10 mL), the calculated risk exceeds the acceptable limit by multiple orders of magnitude.

**Figure 8:**
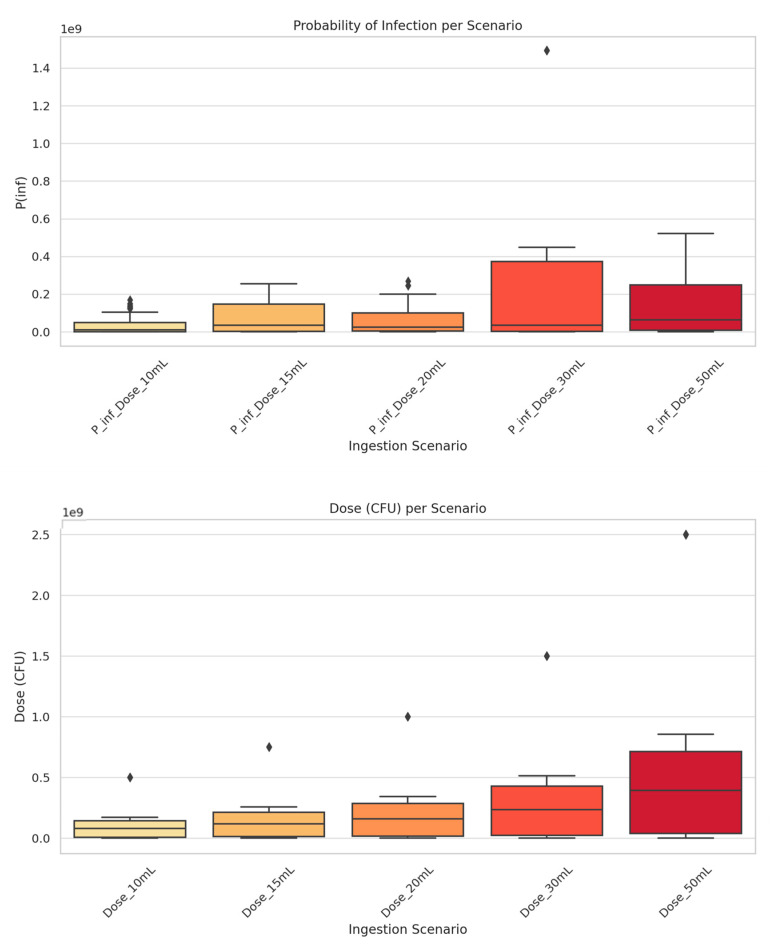
*E. coli* dose–response results per scenario *. * 10 mL: Low-exposure scenario (e.g., splashing or incidental contact), 15 mL: Elderly-specific scenario (moderate intake with increased susceptibility), 20 mL: Children-specific scenario (higher susceptibility and behaviour-specific intake), 30 mL: Moderate-exposure scenario (e.g., swimming), 50 mL: High-exposure scenario (e.g., swimmers with full head immersion). Rhombus symbols indicate outliers.

Τhe double boxplot diagram (Figure 8) illustrates the distribution of microbial doses of *E. coli* in CFU and their corresponding infection probabilities (Pinf) for the five ingestion scenarios examined in the QMRA. The doses were derived from the product of the *E. coli* concentration (C), the ingestion volume (V), and the virulence fraction (IF = 0.01). Concurrently, the infection probability was calculated based on the beta-Poisson dose–response model with parameters α = 0.21 and β = 2.11 × 10^6^, as per [60]. (Supplementary Material S5 presents all the numerical performance of the dose–response relationship for five exposure scenarios).

The box plots illustrate the distribution of microbial risk and ingested dose across different scenarios. The probability of infection gradually increases with the ingested volume; for the 30 mL and 50 mL scenarios, the median values are notably higher, with some reaching or exceeding a 40% chance of infection. While smaller volumes (10–20 mL) carry a lower risk, the probability of infection remains non-negligible.

The analysis also confirms that the ingested microbial dose directly correlates with the volume consumed. In the 50 mL scenario, the highest values reached or surpassed 2.5 × 10^9^ CFU, indicating a potentially massive number of ingested pathogens per exposure. This confirms the fundamental role that the ingested volume plays in determining microbial risk (Supplementary Material S6. Heatmap of mean annual infection risk by scenario and location).

The Infectious Fraction (PRCC_IF) is the most decisive parameter, with consistently high coefficients (0.79–0.82) across all scenarios and regions (Supplementary Material S7. PRCC per scenario and location). This means the variability in *E. coli*’s ability to infect significantly affects the model’s sensitivity. *E. coli* concentration (PRCC_*Ecoli*) also plays a role, with values ranging from 0.37 to 0.41, showing slight variations between scenarios and locations. This variability consistently and substantially contributes to the final risk assessment.

**Figure 9:**
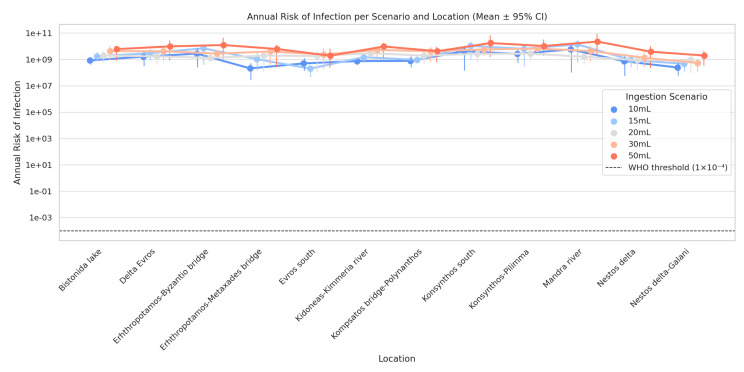
Uncertainty analysis of annual infection risk across ingestion scenarios and sampling locations.

A remarkable effect is recorded regarding the amount of ingestion. Its values are comparable to *E. coli* concentration, demonstrating that exposure habits or behaviours (e.g., age, activity) substantially contribute to the microbial risk. Figure 9 represents the uncertainty analysis of the estimated annual risk of *E. coli* infection, derived from 10,000 Monte Carlo simulation runs for each exposure scenario (10–50 mL) and each sampling location. The representation is performed through the mean and the 95% confidence interval (95% CI), thus specifying both the central estimate and the range of uncertainty of the results.

Uncertainty analysis demonstrated a transparent and predictable increase in microbial risk with greater water consumption, confirming the theoretical relationship described by the dose–response model. In the low exposure scenarios (10–15 mL), the average annual risk in most areas was above the WHO acceptable limit (1 × 10^−4^), indicating a manageable yet non-negligible level of risk. In contrast, moderate and high exposure scenarios (30–50 mL) recorded a significant risk increase, with several areas exceeding the 1 × 10^−3^ limit and, in some cases, reaching or exceeding 1 × 10^−2^ (a contamination rate of >1% per year).

The areas of Mandra River, Kompsatos bridge–Polynanthos, Konsynthos south, and Erhthropotamos–Byzantio bridge emerged as critical microbial risk zones, consistently showing elevated values even in intermediate ingestion scenarios (20–30 mL). These areas also displayed the widest confidence intervals (95% CI), reflecting greater data variability.

## 4. Discussion

### 4.1. Seasonal and Spatial Dynamics of Microbial Indicators

This study confirms the well-established seasonal pattern of *E. coli* distribution in Greek swimming beaches, with peak concentrations observed during the summer months, which is attributed to local climate and anthropogenic pressures from tourism and agriculture [27,28]. Our findings also revealed notable seasonal patterns in microbial contamination. *Clostridium perfringens* spores were most frequent during colder months, consistent with their persistence under low-temperature conditions. In contrast, *Salmonella* spp. was detected sporadically in riverine samples, indicating potential but limited pathogenic contamination. Overall, the highest *E. coli* concentrations were observed in summer, likely reflecting the combined effects of elevated temperatures, reduced river flows, and increased human activity, which together contribute to an increased microbial load during this period. While the presence of *Salmonella* spp. was detected in three seasons, our analysis found no stable predictive relationship with other microbial indicators [69,70,71,72]. The positive statistical association observed between *E. coli* and *Clostridium perfringens* suggests these indicators share common contamination sources. Conversely, the lack of a statistically significant correlation between *E. coli* and *Enterococci* concentrations suggests that distinct local or intra-seasonal factors are more likely to drive the fluctuations of these two key indicators.

Permutation importance analysis from ML models revealed enterococci as the most critical factor for predicting pathogen concentration, while the significant seasonal variation in total coliforms highlights a strong link between environmental variables, such as temperature, and microbial load [73]. The detection of *E. coli* and total coliforms in the Nestos and Evros deltaic ecosystems underscores the role of coastal waters as potential reservoirs for transmitting antimicrobial resistance genes (ARGs) and pathogenicity [74,75,76,77], a matter of global health significance. While our study did not perform molecular detection of pathogenic genes, the confirmed presence of *E. coli* in these ecosystems, which are influenced by agricultural and livestock activities, supports the hypothesis of population exposure to pathogenic strains [78]. These findings emphasize the implications of the One Health framework, as contamination in riverine environments reflects the interconnectedness of human, animal, and environmental health, highlighting that water resources are critical reservoirs and transmission routes for bacteria, including those with antibiotic resistance [79,80].

Beyond water, sand can act as a pathogen reservoir, particularly in summer, due to higher temperatures. This aligns with our findings of increased microbial loads, supporting the hypothesis that the summer season poses an elevated risk from both water and beach environments [81,82]. *E. coli*’s ability to form biofilms and resist stress enhances its survival in sand, contributing to overall microbial load through re-suspension [83,84,85,86].

Spatiotemporal analyses of fecal indicators highlight the critical role of bathing water and sediments. Their concentrations are influenced by environmental factors like rainfall and temperature [8,87]. Therefore, future predictive models should account for microorganism dispersion in sand, as it serves as a secondary reservoir, especially in deltaic areas. Prior research has used regression models to predict *E. coli* concentrations, and the increasing use of ensemble machine ML, like those in our study (RFR and GBR), underscores their superiority for microbial quality prediction [88,89]. Our findings align with other studies that have shown associations between *E. coli* concentrations and variables like turbidity, precipitation, temperature, and land use, indicating a convergence of results regarding the key environmental drivers of contamination [90]. While the median BOD5 value was generally low (3.0 mg/L O_2_), the observed range (up to 12.1 mg/L O_2_) suggests occasional episodes of increased organic load. Crucially, the moderate statistical correlation between BOD_5_ and both *E. coli* and *Enterococci* concentrations confirms that the dynamics of organic pollution significantly influence fecal contamination dynamics in the riverine system.

Our study confirms that areas with intense human activity, such as agricultural and tourist use, show significantly higher *E. coli* concentrations, reinforcing the link between microbial load and land use [91]. The complex dispersal and survival of *E. coli* in aquatic environments remain a challenge, highlighting the need for integrated models that account for flow, sediment, and transport dynamics from various sources [92,93,94,95]. The findings also align with broader environmental challenges in Greece, as highlighted by the OECD, which points to the need for improved water quality monitoring, especially in areas affected by agriculture, urban runoff, and tourism [96]. This is particularly critical in floodplains, where the redistribution of microorganisms from diffuse sources can increase microbial load [96,97]. Our research supports the use of predictive models for decision-making, given the increasing vulnerability of the region to climate change.

The high predictive accuracy of our GBR model (R^2^ = 0.91, RMSE = 7.29) confirms the effectiveness of ML approaches for water quality prediction, consistent with previous studies [98,99,100,101,102,103,104,105,106]. Regarding the predictor variables, the low importance scores for *Salmonella* spp. and *Clostridium perfringens* likely reflect their low prevalence in the sampled waters and their weak statistical correlations with *E. coli* in this dataset. These pathogens may originate from distinct sources or be driven by different environmental factors compared to *E. coli*, thereby limiting their utility as robust predictors in this modelling framework. Conversely, our Permutation Importance analysis identified temperature and seasonality as strong determinants of *E. coli* contamination, corroborating recent findings on the role of climatic variability and extreme rainfall events in shaping microbial water quality [106]. These findings suggest that microbiological concentrations are influenced by multiple factors beyond just geographical location.

### 4.2. QMRA and Prediction Perspectives for E. coli

Water quality is crucial for public health; the WHO reports that its improvement could reduce the global disease burden by approximately 4% [106]. Unsafe water is estimated to contribute to over 2.2 million deaths annually, with a high proportion among children. In the USA, waterborne infection cases are estimated to exceed seven million annually, with recreational water exposure ranking as a significant contributor [106]. Our study’s use of QMRA with Monte Carlo simulation aligns with best practices for addressing the inherent variability and uncertainty in risk assessment [107,108]. Our low-exposure scenario (10 mL) showed a mean annual infection probability of ~0.4%, which is notably lower than risk levels observed in a Norwegian study [109,110].

However, when benchmarked against the WHO reference limit of 1 infection per 10,000 people per year (10^−4^), our findings reveal a substantial microbial risk in certain water bodies and deltaic ecosystems, particularly with regard to recreational use such as swimming. The analysis further underscores the fundamental role of ingested volume in risk determination, as infection probability and microbial dose increased proportionally with exposure. Under the 50 mL scenario, estimated doses reached or exceeded 2.5 × 10^9^ CFU, reflecting a potentially massive pathogen intake per exposure event. The consistently elevated values in scenarios above 30 mL highlight the urgent need for targeted mitigation strategies, especially in areas with persistently high *E. coli* concentrations.

Our higher-exposure scenarios (30–50 mL) recorded a high potential burden, with a mean annual infection probability approaching 11–13% and a maximum of 18% in some areas. This aligns with a meta-analysis showing a significantly increased risk of gastrointestinal infections from coastal water exposure, particularly for activities like swimming [111,112,113,114]. A comparison with a study in South Africa further highlights the variability of risk, with our lower-range values for the 10 mL scenario being similar to their “standard scenario,” while our higher-range values for the 30–50 mL scenarios approached or exceeded their “worst-case scenario” [115]. These differences underscore that risk levels vary significantly depending on geographical area, local microorganism concentrations, and exposure assumptions.

While the overall risk scale can vary depending on the specific pathogen and geographical location, our results are qualitatively consistent with other studies that have applied QMRA to assess *E. coli* risks in aquatic environments in the USA, Peru, and Uganda [52,111,116,117,118,119,120]. Furthermore, our study confirms the international trend of applying and optimizing ML models for environmental predictions, a process that is crucial for understanding and addressing the transboundary transport of microbial pollutants [101,103,104]. The high predictive accuracy of our GBR model (R^2^ = 0.91) aligns with recent research, reinforcing the effectiveness of these algorithms for water quality management [102]. The Permutation Importance analysis identified temperature and seasonality as key factors influencing *E. coli* contamination, a finding corroborated by recent research on the effects of extreme rainfall [105].

Beyond identifying environmental drivers and seasonal patterns, the spatial analysis also highlighted critical risk zones with significant regulatory implications. The areas of Mandra River, Kompsatos bridge–Polynanthos, Konsynthos south, and Erhthropotamos–Byzantio bridge emerged as critical microbial risk zones, consistently showing elevated values even in intermediate ingestion scenarios (20–30 mL). These areas also displayed the widest confidence intervals (95% CI), reflecting greater data variability. A comparison with EU Directive 2006/7/EC revealed potential discrepancies: certain areas classified as “Excellent Quality” based on *E. coli* concentrations may still show an estimated probability of contamination >1 × 10^−3^, especially under high consumption scenarios. This discrepancy could lead to a false sense of security and erode public trust in official water quality assessments. This finding underscores the limitations of relying solely on concentration-based standards without considering exposure pathways and dose–response relationships. Regulatory bodies should reevaluate existing guidelines and integrate QMRA to provide a more holistic understanding of risk. Additionally, transparent communication strategies are needed to explain the potential for higher risk under specific exposure conditions, even in waters classified as “good quality” by current metrics. Ultimately, this approach will guide more targeted interventions and resource allocation, focusing on reducing actual infection risk rather than just meeting indicator thresholds.

Our findings also shed light on the paradox in Greece, where water bodies in protected Natura 2000 areas do not always show better ecological status, indicating potential gaps in existing legal directives [121,122,123]. This highlights the need for studies like ours, which provide quantitative, directly applicable data for policymakers. Finally, our results confirm the international trend of applying and optimizing ML models for environmental predictions, a process that is crucial for understanding and addressing the transboundary transport of microbial pollutants [101,103,104].

### 4.3. Limitations

While this study captures the current potential health risks in the area, a more in-depth analysis would provide a comprehensive understanding of all risks and corresponding mitigation strategies. In the coming year, state and research institutions should integrate the systematic study of pathogens (including protozoa and viruses) detected in aquatic ecosystems. This should be coupled with the detection of physicochemical properties and heavy metals in samples and sediments of the study area. Furthermore, an additional assessment of plant and animal capital and the subsoil will offer a clearer picture of the area’s contamination. Future studies would benefit from a more targeted sampling strategy, specifically designed to capture immediate responses to distinct hydrological events like heavy rainfall or flood episodes, thereby providing a more granular understanding of their impact.

A methodological limitation of this study is the reliance on a single composite sample per site during each seasonal campaign (n = 48 total). This approach, necessitated by the extensive geographical scope, inherently limits the assessment of short-term temporal variability in microbial concentrations, particularly within highly dynamic riverine and deltaic ecosystems. We recommend that future risk assessments in this region integrate temporal field replicates to enhance the statistical robustness and resolution of the data.

Beyond farmers, land workers, and fishermen who contact surface waters in rivers, deltas, and recreational areas, we must also consider populations using swimming waters, seas, and rivers, as well as inbound tourists in the Region, especially during summer. Additionally, integrating more ML models can provide greater accuracy and reliability in future aquatic ecosystem quality studies when combined with risk assessment and management approaches. Therefore, quantifying these risks will lead to more representative mitigation measures and strategic protections for both local residents and tourists throughout the Region of EMT.

## 5. Conclusions

This study successfully demonstrated the presence and spatiotemporal variability of *E. coli* and other microbial indicators in the riverine and deltaic waters of Northeastern Greece. Utilizing an integrated framework of QMRA and ML, we developed a robust predictive model that identified key environmental drivers of contamination. Our permutation importance analysis highlighted that physicochemical parameters along with seasonal and geographical variations significantly affect microbial risk.

Our QMRA analysis revealed a significant, dose-dependent risk of infection from *E. coli* across all examined locations and scenarios. While some areas showed lower values, critical zones such as the Mandra River and the Kompsatos–Polynanthos bridge consistently exhibited a high probability of infection, even under intermediate exposure conditions. These findings confirm the fundamental role of ingested volume and microbial concentration in determining the final risk outcome, demonstrating that even low-volume exposure can lead to a non-negligible risk.

Based on our integrated research, we propose several actionable recommendations to enhance water quality management and protect public health. First, the identified critical microbial risk zones should be designated as priority areas for intensive and targeted monitoring. Secondly, we recommend the integration of predictive ML models into early warning systems to enable proactive risk management and timely public health advisories.

Finally, our findings reveal a significant discrepancy where areas classified as “Excellent Quality” by existing guidelines (e.g., EU Directive 2006/7/EC) can still present a substantial risk of contamination under realistic exposure scenarios. This highlights the limitations of relying solely on traditional criteria. We therefore recommend supplementing these qualitative criteria with QMRA-based approaches to achieve a more comprehensive, health-protective, and meaningful public health assessment. This integrated strategy would not only lead to more representative mitigation measures but also restore public trust by aligning official assessments with the actual health outcomes experienced by the population.

## Figures and Tables

**Table 1:** Descriptive statistics of temperature, pH, total coliforms, *E. coli*, enterococci and BOD_5_ (mg/L O_2_) in all sampling points of East Macedonia and Thrace from 2022 to 2023.

	Temperature (°C)	pH	Total Coliforms (CFU/100 mL)	*E. coli* (CFU/100 mL)	Enterococci (CFU/100 mL)	BOD_5_ (mg/L O_2_)
Mean	15.5	7.49	456.3	134.3	122.9	3.6
Median	14.8	7.46	400.0	78.5	54.5	3.0
St. Deviation	4.4	0.36	244.8	173.3	163.3	2.4
Skewness	0.7	0.30	1.5	1.5	1.8	1.1
Kurtosis	0.9	1.19	2.3	0.8	1.7	1.6
Range	18.7	2.08	1170.0	500.0	500.0	11.3
Minimum	7.2	6.54	130.0	0.0	0.0	0.8
Maximum	25.9	8.62	1300.0	500.0	500.0	12.1

**Table 2:** ML models’ performance metrics (10-fold cross-validation).

ML Model	Root Mean Squared Error (RMSE)	Mean Absolute Error (MAE)	Mean Absolute Percentage Error (MAPE)	Coefficient of Determination (R^2^)%
Random Forest Regressor	12.87	9.06	6.36	93.21
Gradient Boosting Regressor	7.29	4.43	5.22	91.13

## Data Availability

The data presented in this study are available on request from the corresponding author.

## Supplementary Materials

The following supporting information can be downloaded at: https://www.mdpi.com/article/10.3390/toxics13100863/s1, Supplementary Material S1: Ecological and geographic information; Supplementary Material S2: Microbiological and physicochemical protocols; Supplementary Material S3: Modelling parameters as extracted from the Microsoft Azure Studio Classic for Regression machine learning algorithms; Supplementary Material S4: Temporal (a) and spatial (b) variability of physicochemical Parameters (pH, Temperature, BOD_5_) in all sampling locations; Supplementary Material S5: *E. coli* dose-response results per scenario; Supplementary Material S6: Heatmap of mean annual infection risk by scenario and location; Supplementary Material S7: Partial Rank Correlation Coefficients (PRCC) per scenario and location [36,37,38,39,40].
