# Supplementary material for: Quantitative Microbial Risk Assessment of E. coli in Riverine and Deltaic Waters of Northeastern Greece: Monte Carlo Simulation and Predictive Perspectives
Source: Toxics. 2025 Oct 11;13(10):863. doi: 10.3390/toxics13100863 (PMC12567672; doi:10.3390/toxics13100863)
Supplement: Supplementary file 1 [file toxics-13-00863-s001.zip › toxics-3875636-supplementary.pdf]

Supplementary Material

Article

Quantitative Microbial Risk Assessment of *E. coli* in Riverine and Deltaic Waters of Northeastern Greece: Monte Carlo Simulation and Predictive Perspectives

Agathi Voltezou [1], Elpida Giorgi[1], Christos Stefanis [1*], Konstantinos Kalentzis[1], Elisavet Stavropoulou[1], Agathangelos Stavropoulos[2] , Evangelia Nena[3], Chrysoula (Chrysa) Voidarou[4], Christina Tsigalou [1], Theodoros C. Konstantinidis [1], Eugenia Bezirtzoglou[1]

[1] Laboratory of Hygiene and Environmental Protection, Faculty of Medicine, Democritus University of Thrace, Alexandroupolis, Greece; lvoltez@admin.duth.gr (A.V.); elpidagiorgi94@gmail.com; chris.stefanis@gmail.com (CS); kkalentz@med.duth.gr; ctsigalo@med.duth.gr (C.T.); elisabeth.stavropoulou@gmail.com (ES); tconstan@med.duth.gr (T.C.C); empezirt@yahoo.gr (E.B.)

[2] School of Social and Political Sciences, University of Glasgow, Glasgow, United Kingdom; angelostavrop@gmail.com (A.S.)

[3] Laboratory of Social Medicine, Medical School, Democritus University of Thrace, 68100 Alexandroupolis, Greece; enena@med.duth.gr

[4] Department of Agriculture, School of Agriculture, University of Ioannina, 47100 Arta, Greece; x.voidarou@uoi.gr (C.V.)

* Correspondence: chris.stefanis@gmail.com

Supplementary Material S1. Ecological and geographic information

The EMT is geomorphologically characterized by a dualism of mountainous and coastal areas, which is formed by the mountainous mass of southern Rhodope, the mountains of Lekani and Pangaio, forming internal plains and basins as well as coastal lowlands. The Region extends over the southern mountainous mass of the Rhodope mountain range with hilly areas or isolated mountains such as Pangaio and plains near the coastal regions and the mouths of its major rivers, Nestos, Evros and Ardas, which originate in Bulgaria. The main lake of EMT is the Vistonida lagoon, the neighbouring wetlands and lagoons of the Evros Delta, and the artificial lakes of the Nestos, Thisavros and Platanovrissi rivers. The EMT is located in one of the most ecologically sensitive areas of the Mediterranean, including significant cross-border mountain ranges, ecosystems of high ecological and aesthetic value and biodiversity, transnational rivers (Evros, Nestos, Ardas, among others) that flow into bays with wetlands protected by the RAMSAR and extensive coastal areas [1].

Concerning the protected areas found within the EMT, the following should be noted: Within the boundaries of EMT, four (4) National Parks are located: the Dadia-Lefkimmi-Soufli National Forest Park, the Evros Delta National Wetland Park, the National Park of Eastern Macedonia and Thrace and the Rhodope Mountains National Park. Moreover, there are thirty-seven (37) areas of the European Ecological Network Natura 2000, forty-eight (48) areas designated as Wildlife Refuges, (12) in the

[1] https://www.pamth.gov.gr/

prefecture of Evros, 9 in the prefecture of Xanthi, 12 in the prefecture of Rodopi, 7 in the prefecture of Drama and 8 in the prefecture of Rodopi), and twenty-four (24) Landscapes of Outstanding Natural Beauty (LNA). Within the boundaries of the EMT, three (3) areas are located (the virgin Central Rhodope Forest, the virgin Paranesti Forest and the Natural Monument of the Beech Forest in Tsichla-Haydou, Xanthi), which were included in the Network of Biogenetic Reserves of the Council of Europe, with a total area of 1068 hectares. Finally, three (3) Ramsar wetlands are identified: Evros Delta, area 9,267 hectares, code 3GR001; Lake Vistonida, Porto-Lagos, Lake Ismarida and adjacent lagoons, area 24,396 hectares, code 3GR002; Nestos Delta and adjacent lagoons, area 21,930 hectares, code 3GR004[2].

Supplementary Material S2. Microbiological and physicochemical protocols

All water samples were collected in sterile 500 mL containers 20–30 cm below the water surface from locations with an overall sea depth of 0.8–1.3 m. Sampling was typically conducted between 11:30 a.m. and 6:00 p.m., coinciding with periods of peak recreational activity. Immediately after collection, samples were stored and transported at 4 ± 1 °C, and all microbiological analyses were completed within 24 hours to ensure data integrity.

Escherichia coli enumeration followed by membrane filtration using sterile 0.45 μm pore size filters (Pall Corporation). A volume of 100 mL was filtered per sample, and the membranes were then incubated on CM1205B Chromogenic Coliform Agar (OXOID) at 36 ± 2 °C for 21 ± 3 hours. Colonies showing dark blue to violet colouration due to β-D-galactosidase and β-D-glucuronidase activity were recorded as *E. coli*. Confirmatory testing was performed following ISO 9308-1:2014. Results were expressed as colony-forming units per 100 mL (CFU/100 mL) or as log-transformed values ($\log_{10}$ CFU/100 mL) [1].

Intestinal Enterococci were analyzed via membrane filtration under ISO 7899-2:2000. Sterile 0.45 μm filters were used to process 100 mL of each sample, and the membranes were placed on Slanetz and Bartley agar. Plates were incubated at 36 ± 2 °C for 44 ± 4 hours. Typical colonies were counted, and confirmatory testing was conducted using bile esculin azide broth at 44 ± 0.5 °C for 2 hours. Confirmed colonies were considered positive for intestinal Enterococci and reported as CFU/100 mL. [2].

Clostridium perfringens (s. and v. forms) were detected using Lactose Sulfite (L.S.) broth, following enrichment and anaerobic incubation at 46 °C for 18–24 hours. Positive tubes were identified by gas, iron formation and black precipitate. *Clostridium perfringens* detection involved 100 mL of sample filtration through a 0.45 μm membrane, which was then transferred to 9 mL of Lactose Sulfite (L.S.) broth**.** The medium contained 5 g tryptic digest of casein, 2.5 g yeast extract (Difco), 2.5 g NaCl, 2.5 g lactose, 0.3 g L-cysteine HCl per liter of distilled water, pH adjusted to 7.1 ± 0.1. After autoclaving (115 °C for 20 min) and boiling (20 min to reduce oxygen), 0.5 mL of a 1.2% sodium metabisulfite solution and 0.2 mL of a 1% ferric ammonium citrate solution were added. The enriched tubes were serially diluted and incubated aerobically at 46 °C for 24 hours. An aliquot was pre-heated at 80 °C for 20 min before inoculation into L.S. broth to detect spore forms [3].

According to ISO 19250:2010, the detection of *Salmonella* spp. included pre-enrichment, selective enrichment, and isolation on XLD agar. *Salmonella* spp. was detected following ISO 19250:2010 for the microbiological examination of water using culture-

[2] https://www.pamth.gov.gr/images/perifereiako_programma_anaptixis/stratigiki_meleti_perivallontikon_epiptoseon.pdf

based methods. A 10 mL portion of each water sample was transferred aseptically to 90 mL of Buffered Peptone Water (BPW) and incubated at 37 °C for 18–24 hours for non-selective pre-enrichment. Subsequently, 0.1 mL of the pre-enrichment culture was inoculated into 10 mL of Rappaport-Vassiliadis Soya (RVS) broth for selective enrichment and incubated at 42–44 °C for 24–48 hours. Following enrichment, 0.1 mL of each culture was streaked onto Xylose Lysine Deoxycholate (XLD) agar and incubated aerobically at 37 °C for 24 hours. Presumptive *Salmonella* colonies—typically red with black centres—were subcultured onto Nutrient Agar and subjected to further biochemical and serological confirmation tests described in the ISO standard. The final results were reported as the presence or absence of *Salmonella* spp. in 100 mL of sample [4].

Biochemical oxygen demand over five days (BOD_5) was assessed using the dilution method based on the APHA Standard Methods [5] without directly measuring dissolved oxygen. The procedure was carried out in 300 mL glass BOD bottles (Wheaton®) filled with appropriate volumes of surface water samples (50 mL and 150 mL), sterile dilution water, nutrient buffer, nitrification inhibitor (allylthiourea), and a microbial inoculum prepared from Polyseed® (Hach Lange). All bottles were tightly sealed and incubated for five days in the dark at 20 ± 1 °C. Seed control bottles were prepared to ensure the viability of the inoculum and to calculate the seed correction factor. The method was quality-assured using glucose-glutamic acid (GGA) standard solution (300 mg/L), with expected values within the acceptable range (198 ± 30.5 mg/L O_2). Final BOD_5 values were derived following the standard calculation method. pH and temperature of water samples were measured in situ using a calibrated portable multiparameter probe (Hach HQ440D with IntelliCAL sensors).

Supplementary Material- S3. Modelling parameters as extracted from the Microsoft Azure Studio Classic for Regression machine learning algorithms.

Model Hyperparameters

To optimize the performance and generalization capabilities of the predictive models, the following hyperparameters are considered. While the initial run used default settings, a refined analysis would involve a systematic search for optimal parameter combinations (e.g., via GridSearchCV or RandomizedSearchCV). The ranges and descriptions below represent common best practices and suitable starting points for hyperparameter tuning.

1. Gradient Boosting Regressor (GBR)

Gradient Boosting builds an ensemble of weak learners (typically decision trees) sequentially, with each tree correcting the errors of its predecessors.[1] Key hyperparameters include:

- **n_estimators**: The number of boosting stages (i.e., the number of trees to be built).
    - *Default:* 100
    - *Typical Range:* 50 to 500+
    - *Note:* A higher number of estimators can lead to better performance but increases computational cost and the risk of overfitting, especially if not paired with a small learning_rate.
- **learning_rate**: Shrinks the contribution of each tree.

    - *Default:* 0.1
    - *Typical Range:* 0.001 to 0.3
    - *Note:* A smaller learning rate requires a larger n_estimators but generally makes the model more robust to overfitting and often yields higher accuracy.
- **max_depth**: The maximum depth of the individual regression estimators (trees).
    - *Default:* 3
    - *Typical Range:* 2 to 10
    - *Note:* GBR typically performs well with relatively shallow trees, as deeper trees can lead to overfitting.
- **subsample**: The fraction of samples to be used for fitting the individual base learners. (Stochastic Gradient Boosting).
    - *Default:* 1.0 (uses all samples)
    - *Typical Range:* 0.5 to 1.0
    - *Note:* Values less than 1.0 introduce randomness, which can reduce variance (overfitting) and speed up computation.
- **loss**: The loss function to be optimized.
    - *Default:* 'squared_error' (for regression, equivalent to 'ls')
    - *Other Options:* 'absolute_error', 'huber'
    - *Note:* 'squared_error' is standard. 'absolute_error' is more robust to outliers, while 'huber' is a combination of both.

Example of GBR parameters for optimized performance:

Python
```
gbr = GradientBoostingRegressor(
    n_estimators=200,      # Increased number of trees
    learning_rate=0.05,    # Reduced learning rate
    max_depth=4,           # Slightly increased tree depth
    subsample=0.8,         # Enabled subsampling
    random_state=42        # For reproducibility
)
```

2. Random Forest Regressor

Random Forest is an ensemble learning method that constructs a multitude of decision trees at training time and outputs the average prediction of the individual trees[2] to improve accuracy and control overfitting. Key hyperparameters include:

- **n_estimators**: The number of trees in the forest.
    - *Default:* 100
    - *Typical Range:* 50 to 500+

    - *Note:* More trees generally lead to better performance up to a certain point, after which gains become marginal, but computational cost increases.
- **max_features**: The number of features to consider when looking for the best split.
    - *Default:* 1.0 (uses all features)
    - *Typical Options:* 'sqrt' (square root of the total number of features), 'log2' (log base 2), or an integer/float.
    - *Note:* Setting max_features to 'sqrt' is a common and often effective choice for regression, as it introduces randomness and decorrelates the trees, improving ensemble performance.
- **max_depth**: The maximum depth of the tree.
    - *Default:* None (meaning nodes are expanded until all leaves are pure or until all leaves contain less than min_samples_split samples).[3]
    - *Typical Range:* 5 to 30+ (depending on dataset complexity).
    - *Note:* Specifying a maximum depth can help control overfitting, though Random Forests are inherently robust due to ensembling.
- **min_samples_leaf**: The minimum number of samples required to be at a leaf node.
    - *Default:* 1
    - *Typical Range:* 1 to 20
    - *Note:* Increasing this value can smooth the model and prevent the creation of highly specific leaves that might lead to overfitting.
- **min_samples_split**: The minimum number of samples required to split an internal node.
    - *Default:* 2
    - *Typical Range:* 2 to 20
    - *Note:* Similar to min_samples_leaf, increasing this value can prevent the model from learning overly specific patterns.

Example of RF parameters for optimized performance:

Python
```
rf = RandomForestRegressor(
    n_estimators=300,      # Increased number of trees
    max_features='sqrt',   # Feature selection for splitting
    max_depth=10,          # Limited tree depth
    min_samples_leaf=5,    # Minimum samples required at a leaf node
    random_state=42        # For reproducibility
```

One-Sample Kolmogorov-Smirnov Test

	TEMPERATURE (°C)	pH	Total Coliforms (cfu/100mL)	E. coli (cfu/100mL)	Enterococci (cfu/100mL)	BOD5 (mg/L O?)
N	48	48	48	48	48	48

Normal Parameters[a,b]	Mean	15,477083	7,487917	456,27	134,29	122,88	3,595833
	Std. Deviation	4,3568112	,3612270	244,773	173,283	163,269	2,4158130
Most Extreme Differences	Absolute	,151	,068	,260	,276	,298	,217
	Positive	,151	,064	,260	,276	,298	,217
	Negative	-,134	-,068	-,137	-,219	-,226	-,124
Kolmogorov-Smirnov Z		1,050	,468	1,803	1,915	2,061	1,503
Asymp. Sig. (2-tailed)		,221	,981	,003	,001	,000	,022

a. Test distribution is Normal.

b. Calculated from data.

Supplementary Material S4. Temporal (a) and spatial (b) variability of physicochemical Parameters (pH, Temperature, BOD_5) in all sampling locations.

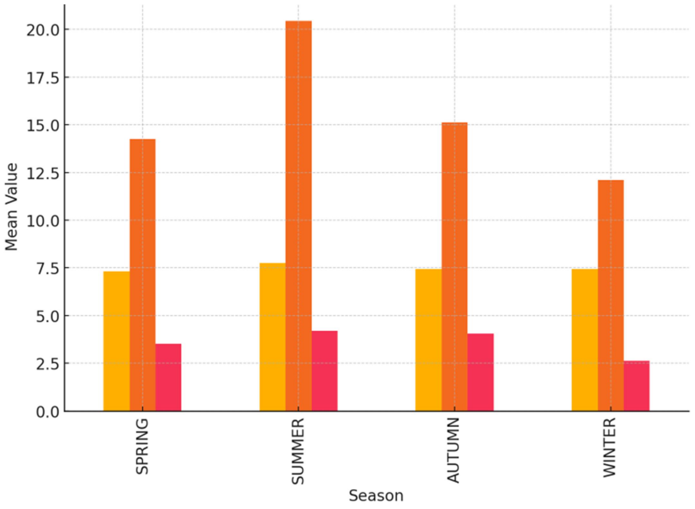

(**a**)

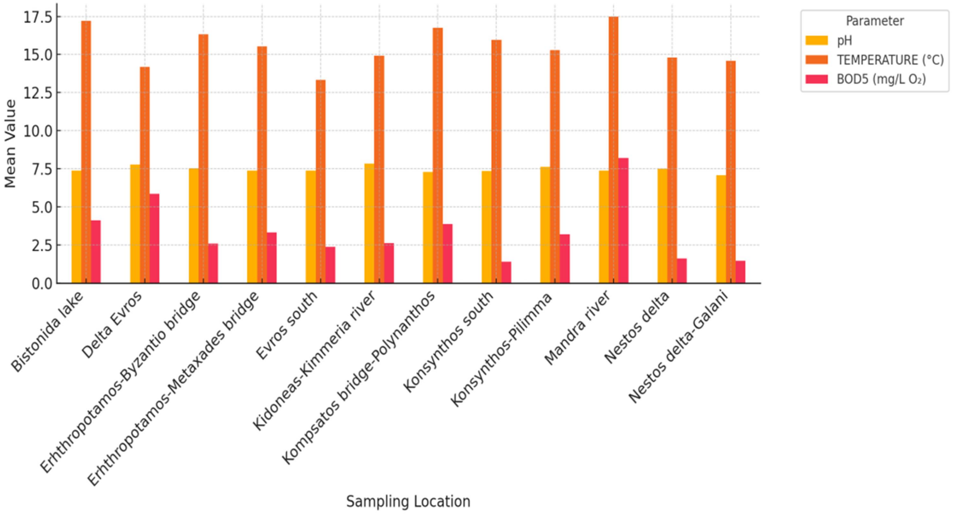

(**b**)

)

The graph below illustrates the percentage presence of *Clostridium perfringens* (v. and s. forms) and *Salmonella* spp. by season.

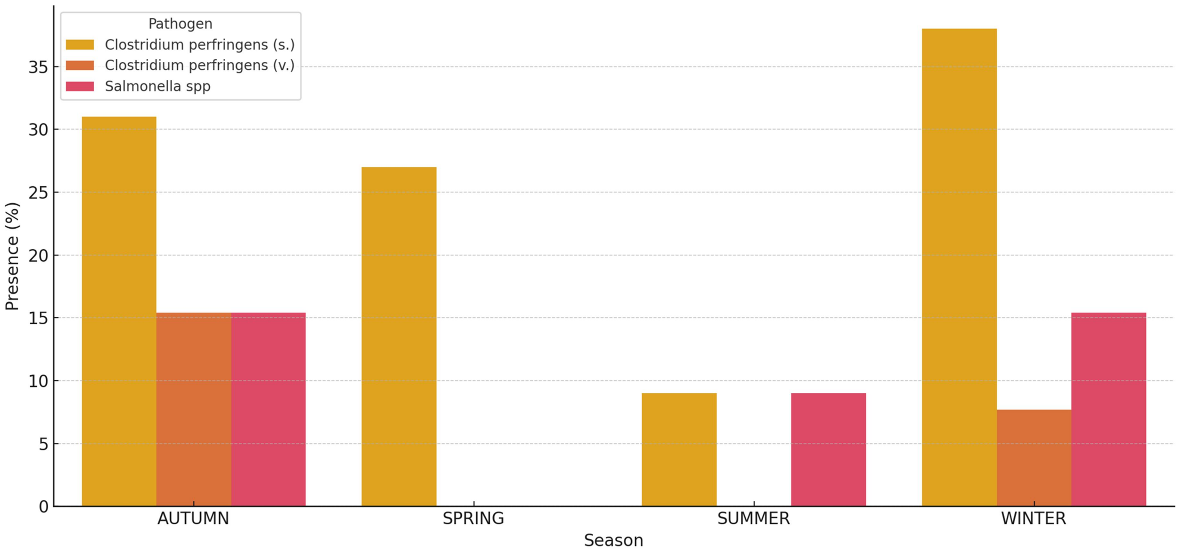

Figure 4. Seasonal distribution of *Clostridium perfringens* (v. and s. forms) and *Salmonella* spp.

Figure 4 illustrates that *Clostridium perfringens* (spore form) exhibits the highest detection frequency in winter (38.5%) and autumn (31%), with the lowest rates in summer (9%). Conversely, the vegetative form of *Clostridium perfringens* was detected to a lesser extent, showing maximum presence in autumn (15.4%) and minimal or zero presence in spring and summer. *Salmonella* spp. detection was observed mainly in winter and autumn (15.4%), with no presence in spring. These findings collectively indicate a more substantial presence of pathogens during the colder months, likely linked to hydrological, temperature, or anthropogenic effects in the corresponding seasons.

Figure 5 represents the percentage of presence of the pathogens *Clostridium perfringens* (v. and s. forms) and *Salmonella* spp. for each sampling location.

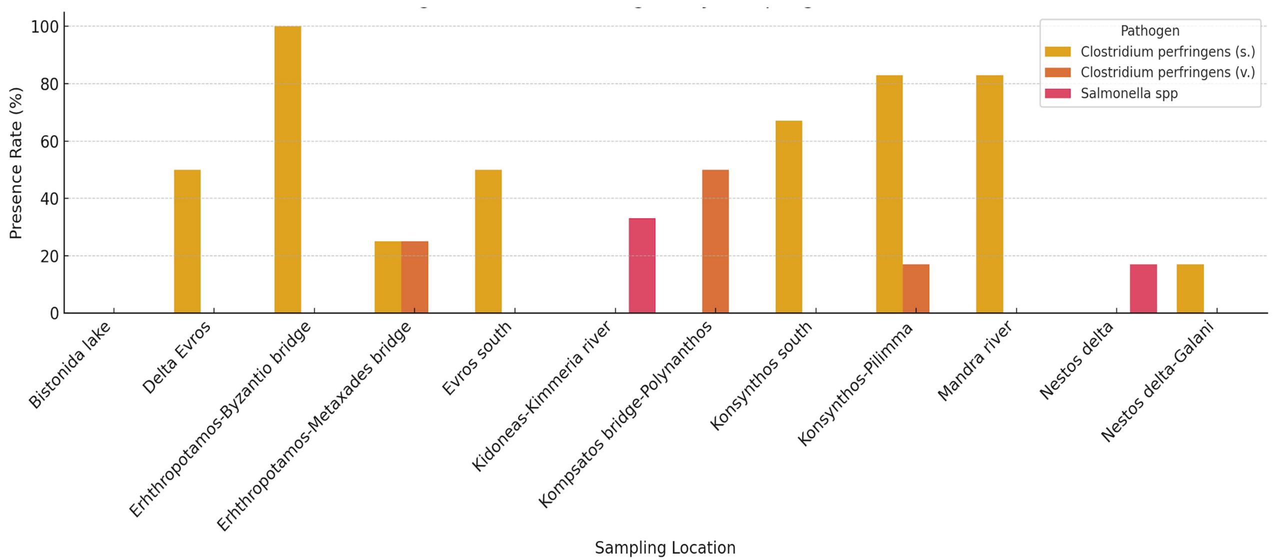

Figure 5. Spatial distribution of *Clostridium perfringens* (v. and . forms) and *Salmonella* spp.

Clostridium perfringens (spore form) was recorded with particularly high presence rates in four areas: the Erhthropotamos-Byzantio bridge (100%), Konsynthos-Pilimma (83%), Mandra River (82%), Konsynthos south (67%), Evros south (51%), and Evros Delta (50%). Correspondingly, the vegetative forms of the microorganism were detected mainly at the Erhthropotamos-Metaxades bridge (25%) and the Kompsatos bridge-Polynanthos (50%). *Salmonella* spp. presented lower overall rates of spatial distribution in the sampling areas, with a maximum value (~35%) in the Kidones-Kimmeris river and lower rates in the Nestos Delta. In conclusion, pathogen distribution is spatially heterogeneous, with specific areas showing a higher presence, mainly for *Clostridium perfringens* (s.).

Supplementary Material S5. *E. coli* dose-response results per scenario *. S5 presents all the numerical performance of the dose-response relationship for five exposure scenarios (10, 15, 20, 30 and 50 mL), according to the Beta–Poisson equation with parameters $\alpha = 0.21$ and $\beta = 2.11 \times 10^6$ (Haas et al., 1999; Tyagi & Kumar, 2021). For each scenario, both the calculated microbial doses (in CFU) and the corresponding infection probabilities per event (P_inf) were recorded.

Dose_ 10mL	Dose_ 15mL	Dose_ 20mL	Dose_ 30mL	Dose_ 50mL	P_inf Dose_ 10mL	P_inf Dose_ 15mL	P_inf Dose_ 20mL	P_inf Dose_ 30mL	P_inf Dose_ 50mL

2E-02	4E-02	5E-02	7E-02	1E-01	2E+06	4E+07	5E+06	7E+05	1E+08
7E-03	1E-02	1E-02	2E-02	4E-02	7E+05	1E+07	1E+07	2E+06	3E+06
1E-01	2E-01	2E-01	3E-01	5E-01	1E+08	2E+08	2E+06	3E+07	5E+08
1E-01	2E-01	2E-01	4E-01	6E-01	1E+07	2E+07	2E+08	4E+06	6E+07
8E-03	1E-02	2E-02	2E-02	4E-02	8E+04	1E+06	2E+07	2E+07	4E+06
4E-02	5E-02	7E-02	1E-01	2E-01	3E+06	5E+06	7E+06	1E+08	2E+08
2E-01	2E-01	3E-01	5E-01	8E-01	1E+08	2E+08	3E+07	4E+08	7E+07
1E-01	1E-01	2E-01	3E-01	5E-01	1E+07	1E+08	2E+08	3E+08	5E+08
1E-03	2E-03	2E-03	3E-03	5E-03	1E+05	1E+05	2E+06	3E+06	5E+05
5E-01	8E-01	1E+00	2E+00	3E+00	5E+07	7E+07	1E+08	1E+09	2E+08
5E-01	8E-01	1E+00	2E+00	3E+00	5E+07	7E+07	1E+08	1E+09	2E+08
5E-01	8E-01	1E+00	2E+00	3E+00	5E+07	7E+07	1E+08	1E+09	2E+08
5E-01	8E-01	1E+00	2E+00	3E+00	5E+07	7E+07	1E+08	1E+09	2E+08
1E-01	2E-01	3E-01	4E-01	7E-01	1E+08	2E+08	3E+08	4E+07	7E+07
5E-02	7E-02	9E-02	1E-01	2E-01	4E+06	7E+07	9E+06	1E+08	2E+07
5E-01	8E-01	1E+00	2E+00	3E+00	5E+07	7E+07	1E+08	1E+09	2E+08
9E-02	1E-01	2E-01	3E-01	5E-01	9E+05	1E+07	2E+07	3E+08	5E+07
2E-01	3E-01	3E-01	5E-01	9E-01	2E+08	3E+08	3E+07	5E+06	9E+07
2E-02	4E-02	5E-02	7E-02	1E-01	2E+06	4E+07	5E+06	7E+05	1E+08
5E-01	8E-01	1E+00	2E+00	3E+00	5E+07	7E+07	1E+08	1E+09	2E+08

2E-01	2E-01	3E-01	5E-01	8E-01	1E+08	2E+08	3E+07	4E+08	7E+07
1E-01	2E-01	2E-01	4E-01	6E-01	1E+08	2E+08	2E+08	4E+08	6E+07
7E-02	1E-01	1E-01	2E-01	3E-01	7E+05	1E+07	1E+08	2E+08	3E+08
9E-02	1E-01	2E-01	3E-01	5E-01	9E+05	1E+08	2E+07	3E+07	5E+08
4E-03	6E-03	8E-03	1E-02	2E-02	4E+05	6E+05	8E+05	1E+06	2E+07
5E-02	8E-02	1E-01	2E-01	3E-01	5E+06	8E+05	1E+08	2E+08	3E+08
2E-02	3E-02	4E-02	5E-02	9E-02	2E+07	3E+07	4E+06	5E+06	9E+06
2E-02	3E-02	3E-02	5E-02	9E-02	2E+07	3E+06	3E+06	5E+06	8E+06
3E-03	5E-03	6E-03	9E-03	2E-02	3E+06	4E+06	6E+05	9E+04	1E+07
1E-01	2E-01	2E-01	3E-01	5E-01	1E+07	2E+08	2E+07	3E+08	5E+07
4E-03	6E-03	8E-03	1E-02	2E-02	4E+05	6E+05	8E+05	1E+06	2E+07
6E-02	9E-02	1E-01	2E-01	3E-01	6E+06	9E+06	1E+08	2E+08	3E+07
1E-03	2E-03	2E-03	3E-03	5E-03	1E+05	1E+05	2E+06	3E+06	5E+05
2E-01	2E-01	3E-01	5E-01	8E-01	1E+08	2E+08	3E+07	4E+08	7E+07
0E+00	0E+00	0E+00	0E+00	0E+00	0E+00	0E+00	0E+00	0E+00	0E+00
6E-02	9E-02	1E-01	2E-01	3E-01	6E+06	9E+05	1E+08	2E+07	3E+07
5E-01	8E-01	1E+00	2E+00	3E+00	5E+07	7E+07	1E+08	1E+09	2E+08
1E-02	2E-02	3E-02	4E-02	7E-02	1E+07	2E+06	3E+07	4E+06	7E+06
7E-03	1E-02	1E-02	2E-02	4E-02	7E+05	1E+07	1E+07	2E+06	3E+06
1E-01	2E-01	3E-01	4E-01	7E-01	1E+08	2E+08	3E+07	4E+07	6E+07

0E+00	0E+00	0E+00	0E+00	0E+00	0E+00	0E+00	0E+00	0E+00	0E+00
0E+00	0E+00	0E+00	0E+00	0E+00	0E+00	0E+00	0E+00	0E+00	0E+00
1E-01	2E-01	3E-01	4E-01	7E-01	1E+08	2E+08	3E+07	4E+07	7E+07
1E-01	2E-01	3E-01	4E-01	6E-01	1E+08	2E+08	3E+07	4E+08	6E+07
0E+00	0E+00	0E+00	0E+00	0E+00	0E+00	0E+00	0E+00	0E+00	0E+00
0E+00	0E+00	0E+00	0E+00	0E+00	0E+00	0E+00	0E+00	0E+00	0E+00
1E-01	2E-01	2E-01	3E-01	5E-01	1E+07	1E+08	2E+08	3E+07	5E+07
5E-01	8E-01	1E+00	2E+00	3E+00	5E+07	7E+07	1E+08	1E+09	2E+08

* 10 mL: Low-exposure scenario (e.g., splashing or incidental contact), 15 mL: Elderly-specific scenario (moderate intake with increased susceptibility), 20 mL: Children-specific scenario (higher susceptibility and behavior-specific in-take), 30 mL: Moderate-exposure scenario (e.g., swimming), 50 mL: High-exposure scenario (e.g., swimmers with full head immersion).

Supplementary Material S6. Heatmap of mean annual infection risk by scenario and location.

The heatmap below depicts the average annual infection risk estimated through Monte Carlo simulations (10,000 iterations) for five exposure scenarios (10–50 mL of water). The values for each sampling area represent the combined effects of *E. coli* concentration, ingestion volume, and infectious fraction.

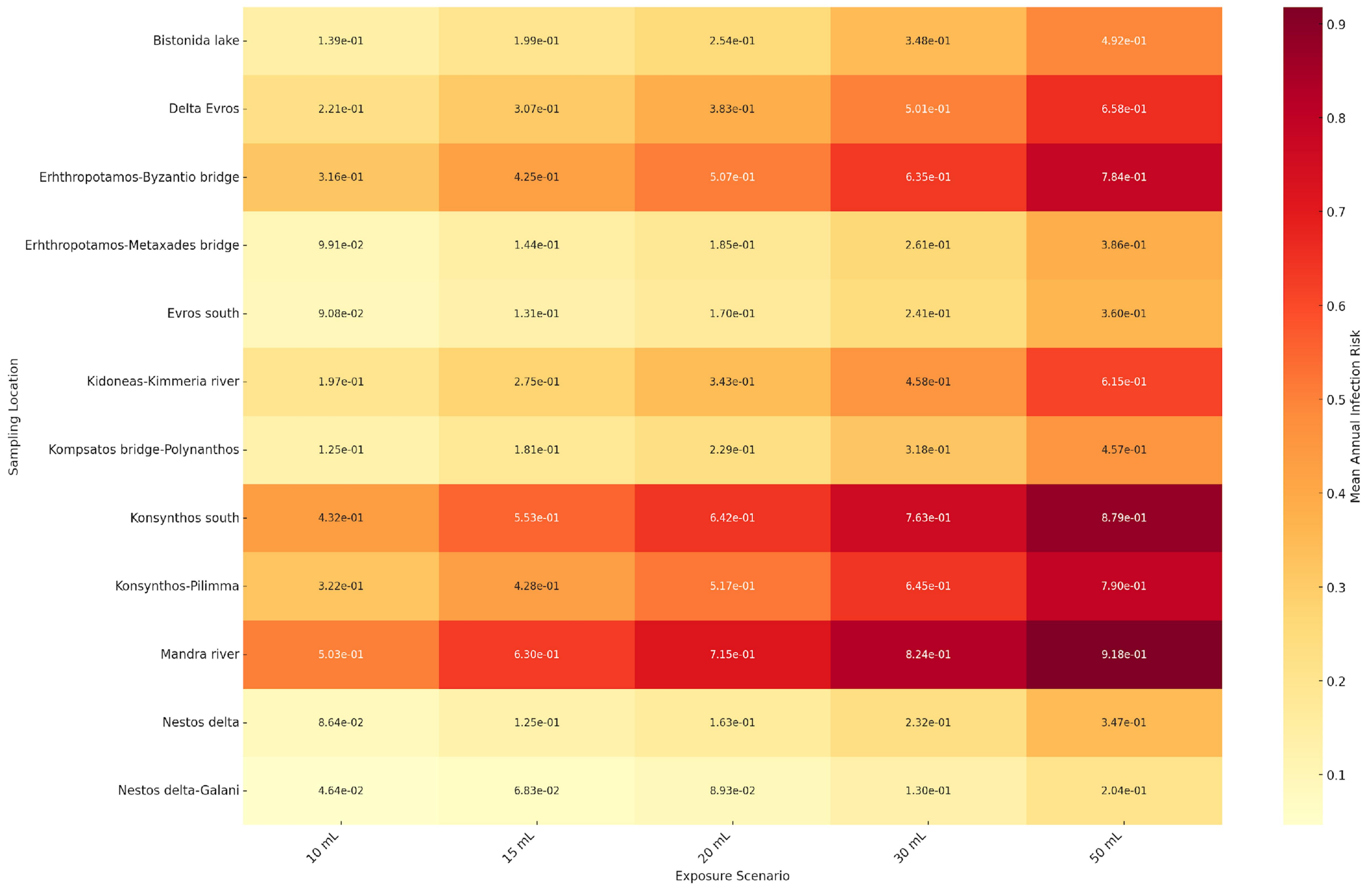

The findings suggest that microbial risk increases proportionally with ingestion, confirming the model's sensitivity to exposure parameters. It's also observed that in all scenarios, most areas far exceed the WHO acceptable risk limit (1.0×10^{-4}). Additionally, locations like Mandra River, Konsynthos south, and Erhthropotamos-Byzantio bridge present the highest values, approaching or exceeding 90% probability of contamination per year in the 50 mL scenario. Conversely, areas with lower values, such as Nestos Delta-Galani and Evros south, record the weakest values but still remain at levels several times higher than the WHO limit. This mapping highlights zones of increased risk, contributing to the prioritization of areas for targeted monitoring and decontamination interventions.

Supplementary Material- S7. Partial Rank Correlation Coefficients (PRCC) per scenario and location. The figure represents the sensitivity analysis conducted using Partial Rank Correlation Coefficients (PRCC) to identify the most influential parameters affecting risk outcomes. These included ingestion volume, E. coli concentration, and the infectious fraction

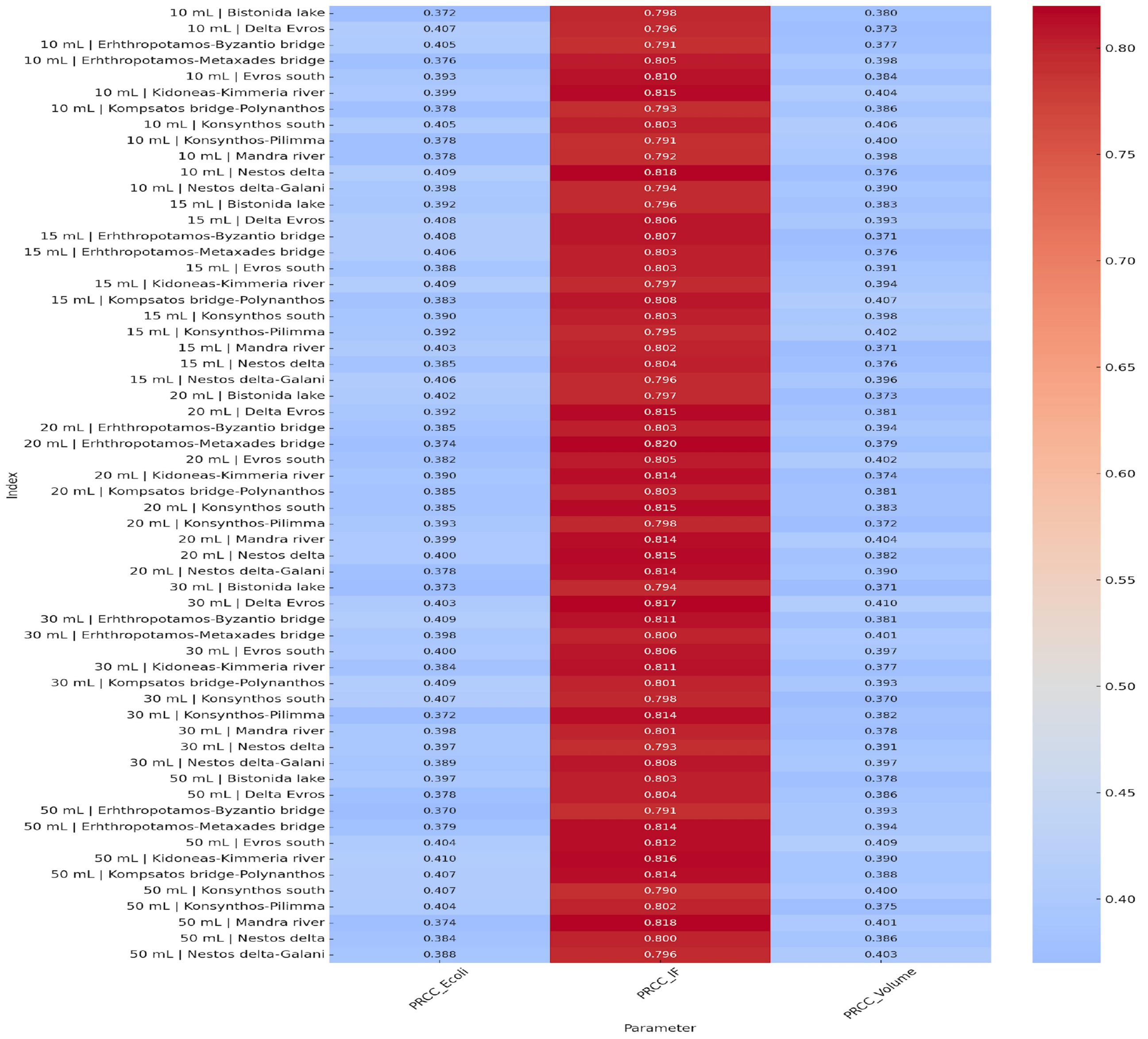

The heatmap presents the partial rank correlation coefficients (PRCC) between the estimated probability of infection (annual risk) and the three critical model parameters per location: PRCC_Ecoli (*E. coli* concentration in CFU/mL), PRCC_IF (infectious fraction of pathogens), and PRCC_Volume (volume of water ingested in mL). PRCC values range from 0 (no correlation) to 1 (strong positive correlation).

References

1. [International Organisation for Standardisation, 2006]. https://www.iso.org/standard/55832.html (accessed on 01 June 2025).

2. [International Organisation for Standardisation, 2000]. https://www.iso.org/obp/ui/#iso:std:iso:7899:-2:ed-2:v1:en (accessed on 01 June 2025).

3. Bezirtzoglou, E.; Dimitriou, D.; Panagiou, A.; Kagalou, I.; Demoliates, Y. Distribution of *Clostridium perfringens* in different aquatic environments in Greece. *Microbiological Research* **1994**, *149*, 2, 129-134. https://doi.org/10.1016/S0944-5013(11)80107-2.

4. [International Organisation for Standardisation, 2010]. https://online.fliphtml5.com/skigm/otwv/?search=nutrient#p=13 (accessed on 01 June 2025).

5. Baird, R.; Bridgewater, L. (2017). Standard methods for the examination of water and wastewater. 23rd edition. American Public Health Association, American Water Works Association, Water Environment Federation.Washington, D.C.
